# Proprotein Convertase 1/3 (PC1/3) in the Rat Alveolar Macrophage Cell Line NR8383: Localization, Trafficking and Effects on Cytokine Secretion

**DOI:** 10.1371/journal.pone.0061557

**Published:** 2013-04-24

**Authors:** Hugo Gagnon, Sarah Refaie, Sandra Gagnon, Roxane Desjardins, Michel Salzet, Robert Day

**Affiliations:** 1 Institut de pharmacologie de Sherbrooke, Université de Sherbrooke, Sherbrooke, Québec, Canada; 2 Université Lille Nord de France, Laboratoire de Spectrométrie de Masse Biologique Fondamentale et Appliquée, EA 4550, Université Lille 1, Villeneuve d’Ascq, France; McGill University, Canada

## Abstract

The proprotein convertase 1/3 (PC1/3) is an important post-translational processing enzyme for the activation of precursor proteins within the regulated secretory pathway. Well characterized for its role in the neural and endocrine systems, we recently reported an unconventional role of PC1/3 as a modulator of the Toll-like receptor innate immune response. There are only a few reports that have studied PC1/3 expression in macrophages, and more investigation is needed to better characterize its function. These studies would greatly benefit from model cell lines. Our study aims to identify and characterize PC1/3 in a relevant model macrophage cell line and to determine the links between PC1/3 and innate immune cellular responses. We describe the rat alveolar cell line, NR8383, as expressing PC1/3 and the most common Toll-like receptors. In NR8383 cells, PC1/3 is localized at the Trans-Golgi network and traffics to lysosome related vesicles upon lipopolysaccharide stimulation. Moreover, we report the co-localization of PC1/3 and Toll-like receptor 4 upon lipopolysaccharide stimulation. Down regulation of PC1/3 by shRNA produce a similar phenotype in NR8383 to what we previously reported in isolated peritoneal macrophages. PC1/3 shRNA induced changes in the cellular organization and expression of the specific trafficking regulator RAB GTPase. As a consequence, NR8383 down-regulated for PC1/3, present an abnormal cytokine secretion profile. We conclude that the NR8383 cell line represents a good model to study PC1/3 in macrophages and we present PC1/3 as an important regulator of vesicle trafficking and secretion in macrophages.

## Introduction

Post-translational modifications are important processes that contribute to the biological regulation of proteins. One such modification is the endoproteolysis of precursor proteins, which can lead to activation, inactivation or functional changes [1]. This cleavage process can be extensive or limited to a few bonds by specific convertases and is followed by amino-terminal, internal and carboxy-terminal modification into smaller biologically active polypeptides [2], [3]. Among them, proprotein convertases (PCs) are a family of subtilisin-like serine proteinases encoded by 9 PC subtilisin/kexin genes (*PCSK1* to *PCSK9*) that encoding PC1/3, PC2, furin, PC4, PC5/6, PACE4, PC7, SKI-1/S1P and PCSK9 respectively [4]–[8]. Seven PCs cleave secretory precursors at basic amino acids within the consensus cleavage site R-X-R/K-R**↓** [4]. PC2 and PC1/3 represent a particular subtype of PCs because they operate within the regulated secretory pathway and are sorted into secretory granules. The expression of PC2 and PC1/3 is widely associated with neuroendocrine tissues; however, we previously reported that both of these neuroendocrine-“specific” convertases are also expressed in macrophages and lymphocytes *in vivo* [9] and are highly responsive to pathogen-associated molecular pattern challenge. This study supports other reports that illustrate the atypical expression of PC1/3 in cells of the immune system. In previous studies, PC1/3 has been detected in a human monocyte-derived macrophage cell line [10] and in differentiated macrophages [11]. In a recent study [12], we characterized an innate immune related phenotype for the PC1/3 knockout (KO) mice challenged with lipopolysaccharides (LPS), which triggers a cascade of events following the stimulation of Toll-like receptor 4 (TLR4) [13]–[15]. We identified a massive cytokine response and demonstrated that the T_h_1 pathway is enhanced in PC1/3 KO mice, which is indicative of a pro-inflammatory response. Our data suggested that PC1/3 plays a vital role in the secretion of bioactive proteins from macrophages. Notably, we used electron microscopy to demonstrate a drastic change in the number and morphology of secretion-related vesicles in macrophages derived from PC1/3 KO mice. We concluded that PC1/3 regulates the cytokine response and thus the innate immune response.

Our previous work describes a novel and unconventional innate response control mechanism mediated by the enzyme PC1/3. Imbalance in the innate immune response is associated with many pathophysiologies [16]–[18] and therapies that target the innate immune response are emerging as potential treatment avenues [19]. Additional knowledge in this field would aid in the development of new therapies and may overcome barriers in this field [20]. The role of PC1/3 as a modulator of innate immunity is unexpected because the mechanism of action of this protein was only extensively characterized in neuroendocrine tissues and cells [21], [22]. Although mouse models have proven useful for the study of PC physiology [23], *in vitro* models provide another level of understanding to simply characterize molecular and cellular mechanisms. In return, cellular models must be established to build solid postulates that can be subsequently transferred to *in vivo* models.

Our aim is to examine the role of PC1/3 in innate immunity, establish a cellular model to study innate immunity and determine whether the known cellular biology of PC1/3 is applicable to this specific system. Here, we report that the NR8383 alveolar macrophage cell line models similar features in terms of PC expression when compared to rat-isolated macrophages, where PC1/3 expression levels are high and PC2 is not expressed [9], [11]. NR8383 cells were previously shown to be sensitive to LPS [24], and thus we took this finding a step further by characterizing the expression of the most common TLRs. We established the cellular localization of PC1/3 in NR8383 cells, but also showed that PC1/3 trafficking is affected by LPS stimulation. Using shRNA, we investigated the consequences of PC1/3 down-regulation on vesicle trafficking and cytokine secretion. Our study establishes the rat alveolar NR8383 cell line as a good cellular model to study the role of PC1/3 in the macrophage cellular innate immune response. We describe LPS-regulated PC1/3 trafficking and provide evidence for PC1/3 modulated macrophage activation through molecular trafficking.

## Materials and Methods

### Reagents and Antibodies

UltraPure *E.coli* 0111:B4 LPS was obtained from Sigma-Aldrich. We obtained the Alexa Fluor® 488 donkey anti-rabbit and Alexa Fluor® 546 goat anti-mouse secondary antibodies from Molecular Probes. Anti-mouse and anti-rabbit IgGs coupled to IRDye800 and IRDye680 were obtained from LI-COR Biosciences. The rabbit anti-PC1/3 (Fus) antibody was previously described [25]. The rabbit anti-PC1/3 targeted against the catalytic domain of PC1/3 was provided by CellSignalling Technologies (No. 11914). The other commercially available antibodies used in this study included anti-TLR4 (ProSci No. 49–321), anti-actin (NeoMarkers, Clone ACTN05), anti-TGN46 (Novus Biologicals No. NB110–60520), anti-EEA1 (BD Transduction Laboratories No. 610456), anti-LAMP1 (University of Iowa, Clone H4A3) and the following antibodies from CellSignaling Technology: anti-RAB5 (No. 3547), anti-RAB7 (No.9367), anti-RAB8 (No. 6975), anti-RAB9 (No. 5118), anti-RAB11 (No. 5589) and anti-EEA1 (No. 3288). Non-targeting (NT) shRNAs in the MISSION RNAi pLKO. 1-puro vector were obtained from Sigma-Aldrich. Several shRNA sequences targeting rat PC1/3 (accession number NM_017091) were tested. TRC sequences for shRNAs against human and mouse PC1/3 were modified to target the rat PC1/3 sequence using the Addgene pLKO shRNA design recommendation (see web link below). The most effective sequence used in this paper was 5′-AATTATGACCCAGAGGCTAGC-3′, which targets 21 nucleotides starting at position 757 in the gene and corresponds to a modified version of TRCN0000032922. This sequence was cloned into the pLKO.1 vector using the protocol available at http://www.addgene.org/tools/protocols/pLKO/. We used the following previously described probes: furin [26], PC2 [27], PC1/3 [28], PACE4 [29], PC5/6 [30] and PC7 [31].

### Cell Culture

The rat alveolar macrophage NR8383 cell line [32], [33] was cultured in Ham’s F12K medium supplemented with 15% fetal bovine serum (FBS; Wisent Bioproducts, St Bruno, QC) at 37°C in a humidified atmosphere (5% CO_2_). For LPS stimulation, the cells were starved overnight, the medium was replaced with fresh medium and the cells were stimulated with 100 ng/ml LPS for the indicated times.

### Confocal Microscopy and Co-localization

The NR8383 cells were grown in culture flasks on coverslips and stimulated as described in text. The cells were treated and observed with an Olympus FV1000 inverted confocal laser-scanning microscope as previously described with minor modifications to the protocol [34]. Primary antibodies were incubated in a moist chamber overnight at 4°C. Coverslips were mounted with SlowFade Gold Antifade Reagents (Life Technologies). Olympus Fluoview software (version 1.6b) was used for image acquisition. The images were further processed and analyzed using ImageJ 35,36 with Bio-Formats plugin [37]. Statistical analysis was performed using GraphPad Prism 5 software.

### Western Blot Analysis

After treatment, the cells were detached, centrifuged at 500 g and washed once with ice-cold PBS. The cells were subsequently lysed with radioimmune precipitation assay lysis buffer containing Complete Mini protease inhibitor and processed as previously described [12]. ImageJ software was used to quantify bands and statistical analysis was performed using GraphPad Prism 5 software.

### Northern Blot

Total RNA was extracted from tissues using a guanidium isothiocyanate method that includes a lithium chloride precipitation step [38]. Northern blotting was performed as previously described [9].

### Real-time Quantitative PCR

Total RNA was extracted using the Qiagen RNA isolation kit (Qiagen, Valencia, CA, USA). The quality of the total RNA samples was assessed using an Agilent Bioanalyzer with RNA Nano Chips (Agilent Technologies, Palo Alto, CA, USA). Real-time quantitative PCR reactions were performed as previously described [39]. Briefly, 1 µg of RNA was reverse transcribed, and qPCR analysis reactions were performed using a Stratagene Mx3005P5 instrument. To amplify actin, we used the following primers: forward 5′-GCGTCCACCCGCGAGTACAAC-‘3 and reverse 5′-CGACGACGAGCGCAGCGATA-‘3. To amplify PC1/3, we used the following primers: forward 5′-GGTGAATGTTGTGGAGGAGAAGC-‘3 and reverse 5′-AGCACTTTGTAGGAGCCGTAGC-‘3. To amplify proSAAS, we used the following primers: forward primer (5_-TGCTGCTCTTGGGTCTTCTG-3_), reverse primer (5_-GAGTGCTCGTCTCAGCCAA-3_). The relative expression levels were calculated using β-actin as a reference gene with the formula (1+ amplification efficiency)^−Δ(ΔCT)^. All experiments were performed in duplicate in 3 independent experiments (*n* = 3).

### NR8383 Knockdown using Lentivirus Transduction

Lentiviral particles containing the MISSION RNAi pLKO. 1-puro vector were produced in HEK293FT cells following the manufacturer’s instructions (Sigma-Aldrich, St. Louis, MO, USA). Viral titers were calculated in HT1080 cells using a serial dilution approach. Lentiviral transduction was performed in 12-well plates with a cell density of 5×10^5^ cells/mL and a multiplicity of infection (MOI) of 1.5. After 2 days, the infected cells were selected using growth medium containing 12.5 puromycin/mL. Upon characterization, 2 polyclonal cell populations were selected for further studies to avoid any artifacts associated with individual clone selection and to cross-validate our observations [40].

### ELISA

Cells were plated at densities of 2.5×10^5^ or 5×10^5^ cells/ml in serum-free F12K medium overnight at 37°C in a humidified atmosphere containing 5% CO_2_. The medium was subsequently changed, and the cells were stimulated with vehicle or 100 ng/ml LPS without serum for the indicated times. The medium was collected and cleared of cells and cell debris by centrifugation at 800 g for 5 min. Cytokines were measured using ELISA kits specific for rat TNF-α (BD biosciences), IL-1β and IL-6 (R&D Systems) according to the manufacturers’ instructions. Statistical analysis was performed using GraphPad Prism 5 software.

## Results

### PC1/3 and Most Common TLRs are Expressed in NR8383 Cells

**Figure 1:**
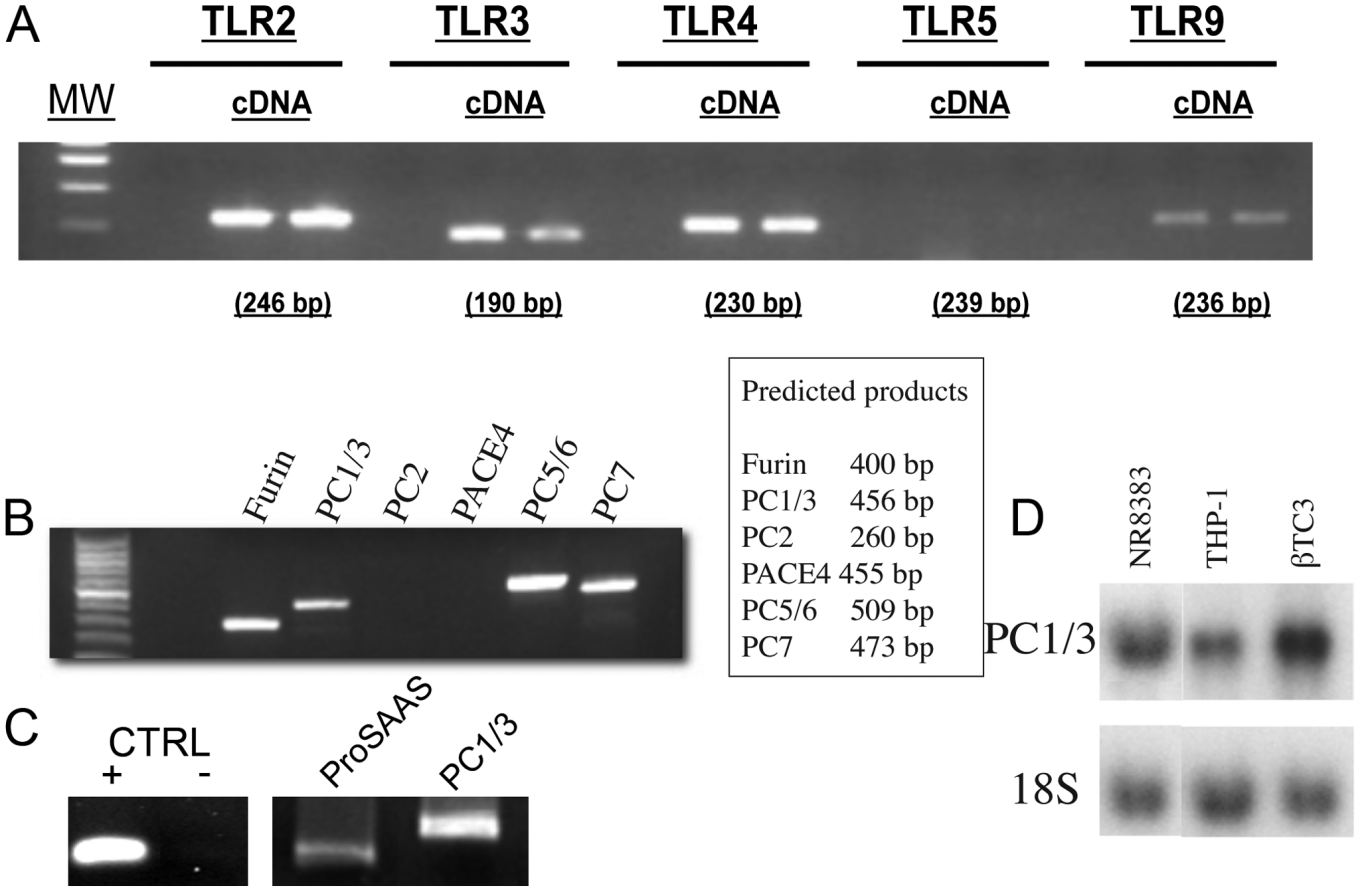
Characterization of the NR8383 cell line. A. Expression of TLR in NR8383 by RT-PCR. B. Expression of PCs in NR8383 by RT-PCR with the expected band length in base pairs (bp). C. Expression of proSAAS and PC1/3 by qRT-PCR. Negative control (CTRL –) is a non template control and positive control (CTRL +) is 10 pg of rat proSAAS cloned into pCDNA3.1 vector. D. Northern blot analysis of PC1/3 in NR8383, PMA-induced THP-1 and control β-TC3.

Although various macrophage cell lines are available, little information was available regarding their PC1/3 expression. The human macrophage cell line THP-1 was shown to express PC1/3, however, THP-1 cells need to be stimulated with phorbol 12-myristate 13-acetate (PMA) to induce PC1/3 expression [10]. We have screened various cell lines and discovered that NR8383 cells, originally derived from rat alveolar macrophages [32], [33], express PC1/3 but not PC2 (**Figure 1B**). We confirmed that the resulting RT-PCR product was PC1/3 by sequencing (**sup. data Figure S1**). This observation agrees with previous data obtained using isolated rat alveolar macrophages and splenic macrophages [11]. Moreover, NR8383 cells were shown to be sensitive to TLR ligands, notably LPS [24] and polyI:C [41], making them a good cellular model in which to examine the role of PC1/3 in TLR-based activation of macrophages. However, previous reports have not addressed which of the most common TLRs are expressed in NR8383. We thus used RT-PCR (**Figure 1A**) to detect the expression of the intracellular TLR receptors TLR3 and TLR9 and the cell surface receptors TLR2 and TLR4. We did not detect the expression of TLR5. To our knowledge, this is the first time that TLR expression profile has been characterized in NR8383 cells. We also used RT-PCR to examine the expression of other PCs and detected the expression of Furin, PC5/6 and PC7 but not PACE4 (**Figure 1B**). Moreover, we tested the expression of proSAAS, a protein that often co-localises with PC1/3 in neural and endocrine cells [28] and may be relevant to regulate the activity of PC1/3. We conducted a qPCR experiment to amplify rat proSAAS and rat PC1/3 and ran the product on gel (**Figure 1C**). Accordingly, proSAAS expression was detected and its expression level was 2.5 times lower than that of PC1/3. We further confirmed PC1/3 expression in NR8383 macrophages using northern blot analysis (**Figure 1D**) compared with the known PC1/3-expressing cells beta-TC3 [42] and PMA-differentiated THP-1 macrophage cells [10].

### PC1/3 Down-regulation by shRNA and PC1/3 Protein Expression in NR8383 Cells

**Figure 2:**
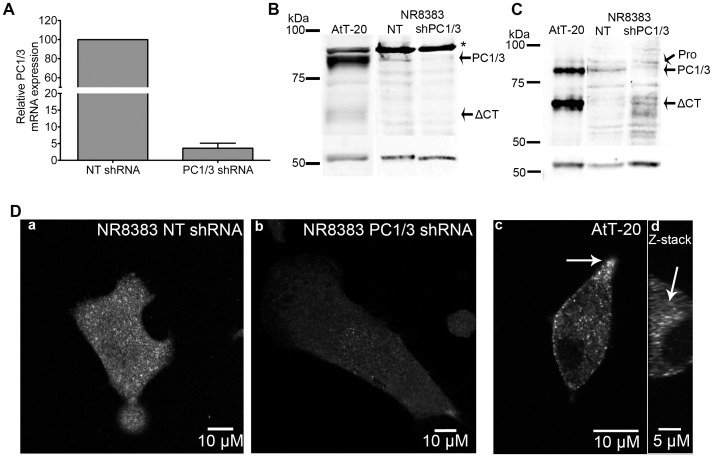
PC1/3 protein expression and down-regulation by shRNA in NR8383 cells. A. qPCR analysis of the relative expression of PC1/3 in NR8383 cells stably transduced with non-target (NT) shRNA and PC1/3 shRNA. Relative expression was normalized on β-actin (*n* = 3). B–C. Western blot of PC1/3 in NR8383 cells stably transduced with NT shRNA or PC1/3 shRNA as well as AtT-20 cells using a PC1/3 antibody targeted against the C-terminal domain (B) or the catalytic domain (C). 15 µg of AtT-20 protein and 50 µg of NR8383 cells were loaded. Pro PC1/3 is detected around 90 kDa, full-length PC1/3 (PC1/3) is detected at 87 kDa and fully C-terminally matured PC1/3 (ΔCT PC1/3) is detected at 66 kDa. C. Confocal microscopy of PC1/3 by indirect immunofluorescence on NR8383 cells expressing PC1/3 shRNA (b) and NT shRNA (a) using anti-PC1/3 directed against the C-terminal domain. A positive control (AtT-20) is shown, which exhibits specific secretory granule-like labeling (c) (3× magnification in the rectangle inset). d- is a Z-stack reconstruction of confocal plane from (c) showing TGN labeling of PC1/3 in AtT-20 cells.

The successful down-regulation of PC1/3 by shRNA was essential in our experiments for two reasons. First, cells that exhibit PC1/3 down-regulation were used as negative controls to ensure the specificity of the PC1/3 antibodies used, and second, these cells were used to determine if NR8383 cells are a comparable model with macrophages isolated from PC1/3 KO in our previous study [12]. We induced stable PC1/3 knockdown using lentiviral delivery of shRNAs. Using this method, we obtained a stable knockdown of PC1/3 using a multiplicity of infection of 1.5, which resulted in a 98% reduction of PC1/3 mRNA expression (**Figure 2A**). We also examined PC1/3 protein levels. The principal antibody used in this study is directed to the C-terminal region of PC1/3. It best recognizes full-length PC1/3, but can to a lesser extent detect the C-terminal matured form of PC1/3 (referred here as ΔCT) (**Figure 2B**). We have previously confirmed the specificity of this antibody [12]. Using 15 µg of an AtT-20 cell protein extract, we detected full-length PC1/3, but also the 66 kDa ΔCT-PC1/3. In NR8383 cells, we detected PC1/3 as a weak band of approximately 87 kDa; however, since a large amount of protein (50 µg) needed to be loaded to achieve this detection, resulting in increased non-specific band detection. The observed 87 kDa band observed corresponds to the full-length form of PC1/3. We previously characterized this band and confirmed its identification by mass spectrometry [12]. We also show that PC1/3 down-regulation in NR8383 cells resulted in the disappearance of the PC1/3 specific band (**Figure 2B**). Using another antibody directed against the catalytic domain of PC1/3 with better sensitivity for the matured forms of PC1/3, we detected pro PC1/3, full-length PC1/3 and ΔCT-PC1/3 using 15 µg of AtT-20 protein extract (**Figure 2C**). However, in NR8383 cells we could only detect a specific PC1/3 protein band corresponding to full-length PC1/3, which disappeared in PC1/3 down-regulated NR8383 cells. It is important to note that different from NR8383 cells, AtT-20 cells have the capacity to accumulate such proteins in secretory granules. Thus the detection of PC1/3 in NR8383 cells under western blotting conditions is not ideal. We therefore turned to indirect immunofluorescence labeling (IF) to further characterize PC1/3 in NR8383 cells. When we performed IF on wild type NR8383 cells (**Figure 2D** **, a**) and on PC1/3 down-regulated NR8383 cells (**Figure 2D** **, b**), we easily observed the consequences of reduced PC1/3 expression. In AtT-20 cells, as expected [43], [44], PC1/3 was accumulated in dense core secretory granules (DSCG) (**Figure 2D** **, c**) and as well as a TGN like region that could be recognized by reconstructing the vertical confocal plane (**Figure 2D** **, d**). These data confirm that there are no equivalent structures as DCSG in NR8383 cells, but also show that PC1/3 is more easily detected with IF than for western blotting and encouraged us to further characterize the intracellular localization of PC1/3 in NR8383 cells.

### PC1/3 Cellular Localization in NR8383 Cells

**Figure 3:**
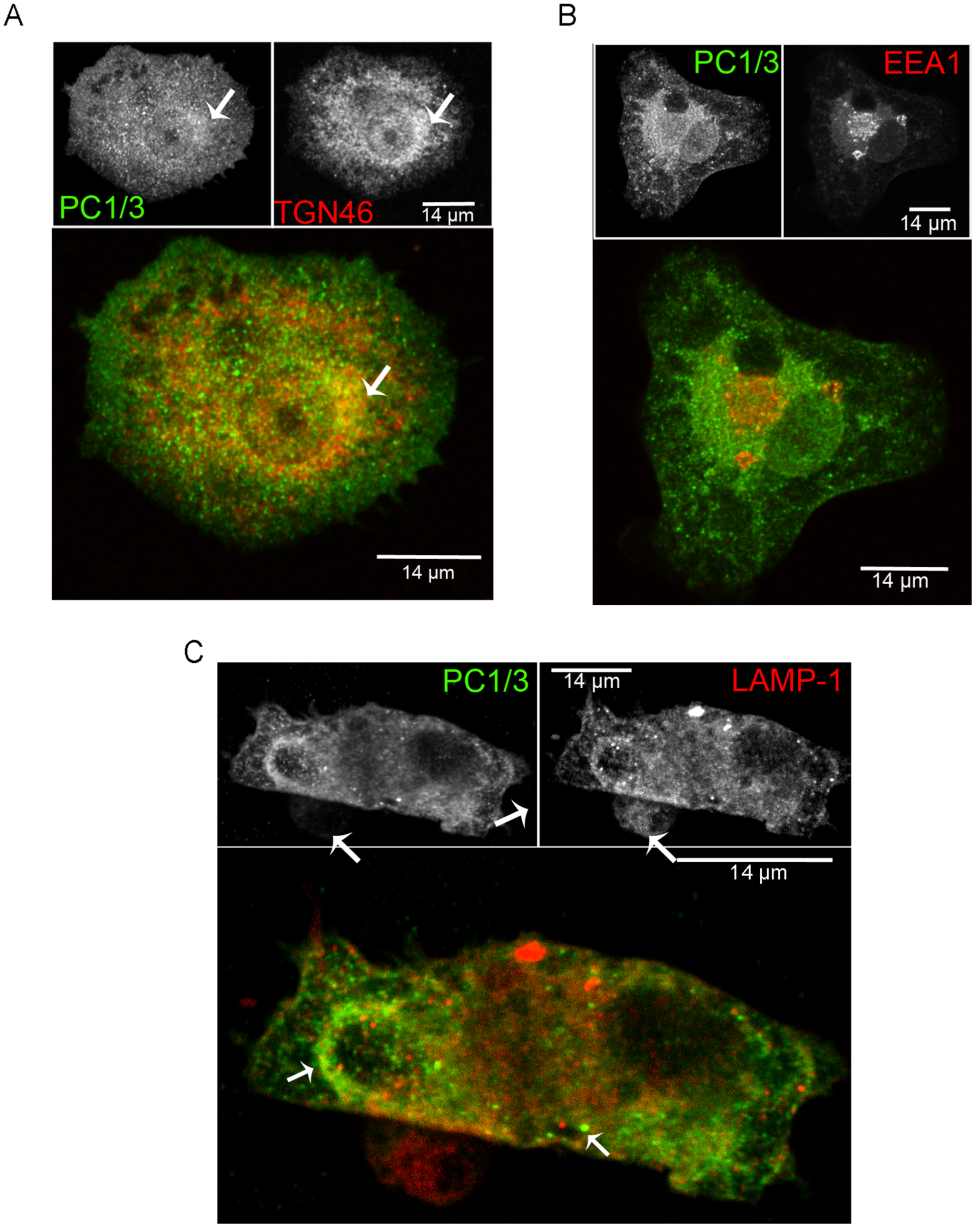
PC1/3 cellular distribution in NR8383 cells as examined by immunofluorescence. Indirect immunofluorescence confocal microscopy with anti-PC1/3 (green), (A) anti-TGN46 (red), (B) anti-EEA1 (red) and (C) anti-LAMP1 (red). A. The arrow indicates the TGN region where PC1/3 and TGN46 co-localize. C. The left arrow indicates the phagocytic structure and the lower right arrow indicates the lysosome-like structure where PC1/3 and LAMP1 are partially co-localized.

**Figure 4:**
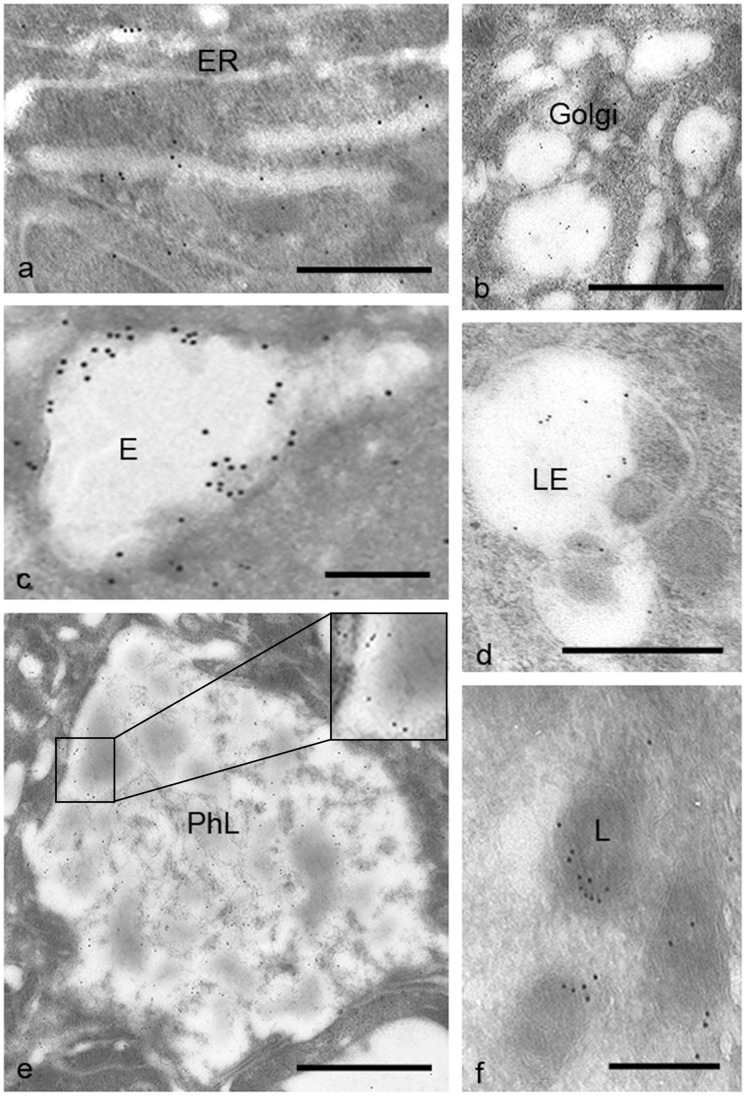
Cellular distribution of PC1/3 in NR8383 determined by immuno-gold electron microscopy. Electron micrographs of NR8383 cells immunolabeled for PC1/3. Gold particles can be observed in: (a) endoplasmic reticulum (ER), bar = 500 nm; (b) Golgi apparatus, bar = 1 µm; (c) endosome (EE), bar = 250 nm; (d) late endosome (LE), bar = 500 nm; (e) phagolysosome (PhL), bar = 1 µm inset is a 2× magnification; (f) lysosomes (L), bar = 250 nm.

In neuroendocrine cells, PC1/3 is typical concentrated in DSCG [43], [44]. In NR8383 cells, PC1/3 mainly accumulated in the Golgi/TGN and shows labeling of vesicle-like structures throughout the cells. To better characterize the intracellular distribution of PC1/3 we carried out IF confocal co-localization studies. Our data shows that PC1/3 is co-localized with TGN46, a common TGN marker [45], [46] (**Figure 3A**). However, there was an additional PC1/3 perinuclear localization, which may represent endoplasmic reticulum (ER) and vesicle-like structures. To identify the vesicle-like structures, we performed further co-localization experiments. Little or no co-localization was observed with the early endosome marker EEA1 [47], [48] (**Figure 3B**). However, PC1/3 was co-localized with LAMP1 (**Figure 3C**) a marker of late endosomes, lysosomes [49] and phagolysosomes [50]. This co-localization was especially observed in forming phagosomal structures. The remaining PC1/3-containing vesicles are most likely recycling endosomes and secretory vesicles. In order to confirm our IF results and further map PC1/3 localization in NR8383 cells, we performed an electron microscopy immunogold labeling study of PC1/3. PC1/3 was observed in the ER and Golgi (**Figure 4a,b**) but also showed extensive accumulation at the periphery of structures corresponding to endosomes (**Figure 4c**), multivesicular structures corresponding to late endosomes (**Figure 4d**) and phagolysosomes (**Figure 4e**) as well as in lysosomes (**Figure 4f**). To our knowledge, this is the first time that PC1/3 localization has been so extensively characterized in non-neuroendocrine cells. It is likely that PC1/3 does not accumulate in specialized secretory granules but instead is retained in the TGN and migrates at least partially to lysosomes or secretory vesicles.

### LPS Induces PC1/3 Trafficking and Co-localization with TLR4

**Figure 5:**
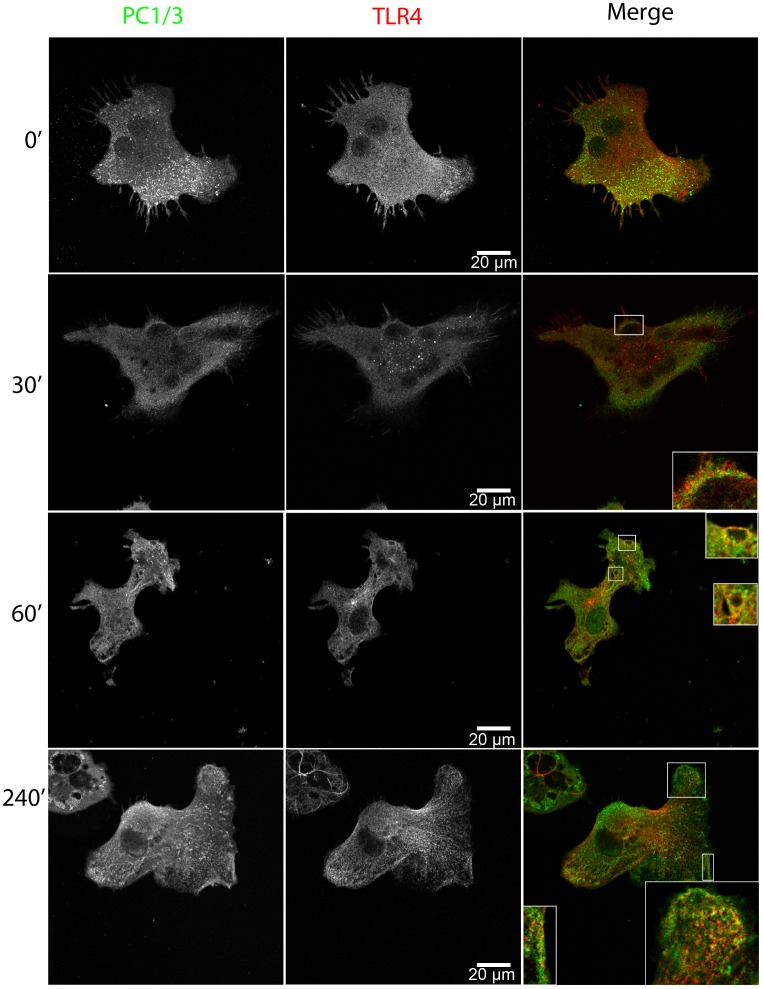
LPS induces PC1/3 trafficking and co-localization with TLR4. Confocal images of NR8383 cells double-labeled with anti-PC1/3 (green) and anti-TLR4 (red) and incubated with 100 ng/ml LPS for the indicated time. Insets represent 3× magnification of regions where PC1/3 and TLR4 show partial co-localization.

Macrophage secretion is regulated differently compared to specialized secretory cells [51], [52] and they secrete several factors, mostly cytokines, following innate immune stimulation. We therefore tested the notion that LPS, which is a TLR4 and gram-negative bacterial-like stimulus, would induce PC1/3 trafficking in NR8383 cells. We observed that LPS induced the time-dependent translocation of PC1/3 toward a phagolysosomal-like structure that co-localized with TLR4 (**Figure 5**). Only 30 min after the initial LPS stimulation, PC1/3 showed good co-localization with TLR4 in these phagolysosomal-like structures, which was sustained over time. After 4 h of stimulation, the increased accumulation of PC1/3 was observed within phagolysosomal structures, suggesting that PC1/3 was being internalized into multivesicular bodies. This observation was also made using a different PC1/3 antibody that recognizes the N-terminus of PC1/3 (**sup. data Figure S2**). It is also worthy to note that PC1/3 accumulation in vesicles near the plasma membrane (PM) was observed after LPS stimulation.

### LPS Stimulation Induces PC1/3 Translocation from TGN46 to LAMP1 Compartments

**Figure 6:**
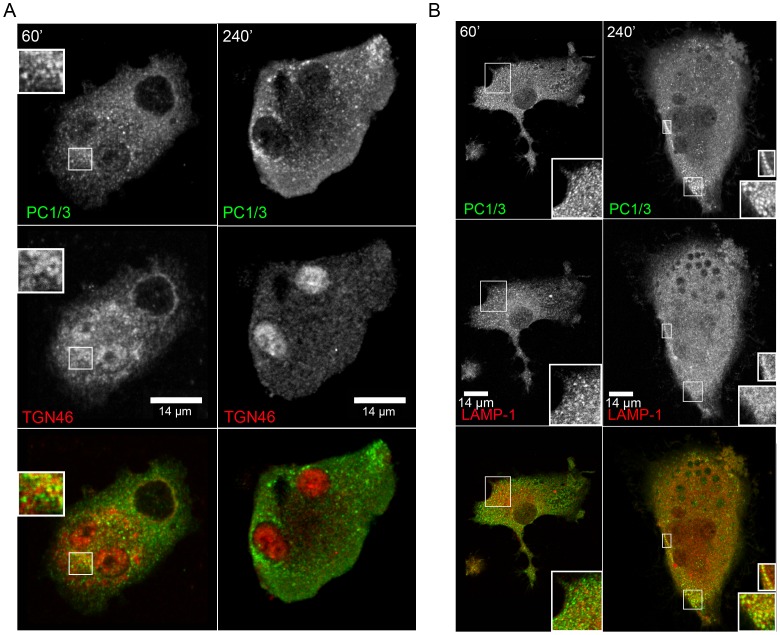
LPS induces PC1/3 trafficking into LAMP1 phagolysosomal structures. Confocal images of NR8383 cells doubly labeled with anti-PC1/3 (green) co-localizing with (A) anti-TGN46 (red) and (B) anti-LAMP1 (red) after 60 and 240 min of stimulation with LPS (100 ng/mL). A. The inset shows the remaining co-localization between TGN46 and PC1/3 after 60 min of stimulation. B. The inset shows a low level of co-localization with LAMP1 after 60 min of LPS stimulation and increased co-localization with LAMP1 after 240 min of LPS stimulation.

We next characterized the LPS-induced PC1/3 trafficking using various organelle markers. After 60 min of LPS stimulation, PC1/3 was still co-localized with TGN46 (**Figure 6A**). We also observed that LPS induced a reorganization of TGN46 such that this marker became more concentrated in the center of a vacuolar structure. PC1/3 was observed to localize to both the middle and the surrounding areas of these structures. After 240 min of LPS stimulation, PC1/3 no longer co-localized with the TGN46 marker but rather localized near the PM and in vesicles surrounding large vacuolar structures. This vacuolar structures correspond to where PC1/3 and TLR4 co-localize. PC1/3 also co-localized with LAMP1 in the vesicles surrounding vacuolar structures only after 240 min of LPS stimulation (**Figure 6B**) but not after 60 mins. These data bring further support to the observation that PC1/3 and LAMP1 are co-colocalized (**Figure 3C**), mostly when phagosomal structures are present. Little or no co-localization of PC1/3 was observed with the early endosome marker EEA1 after 60 and 240 min of stimulation (**sup. data Figure S3**).

The sum of these data indicate that LPS induces PC1/3 trafficking into TLR4-containing structures and that LPS induces the exit of PC1/3 from TGN structures toward lysosomal and phagolysosomal vesicles. The drastic change in the distribution of TGN46 is indicative of TGN reorganization after macrophage activation by LPS, as previously described [53].

### Down-regulation of PC1/3 Disrupts the Spatial Organization of Vesicle Trafficking Markers in NR8383 Cells

**Figure 7:**
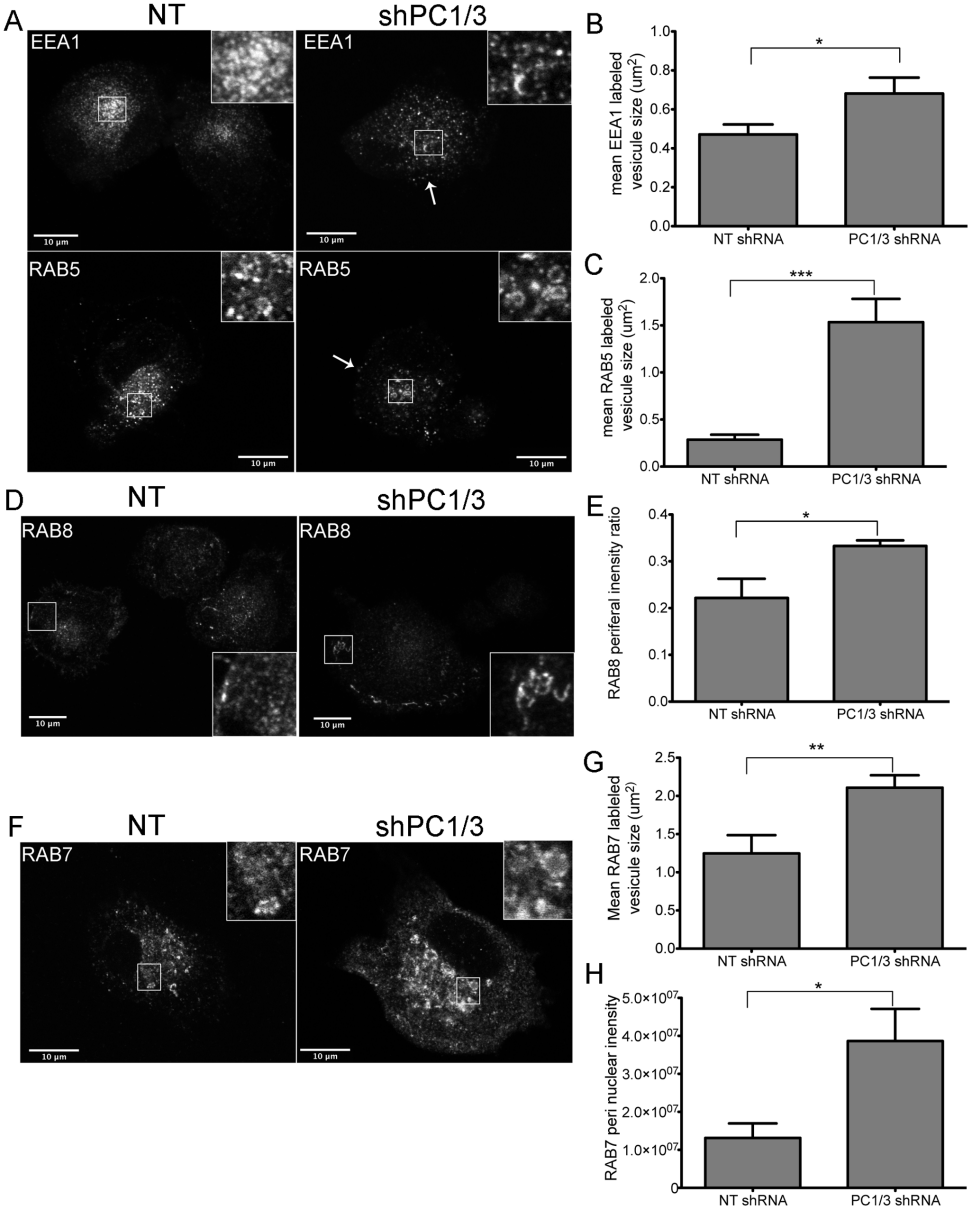
Effects of PC1/3 down-regulation on vesicle trafficking markers. Confocal images of NR8383 cells stably transformed with control shRNA (NT) and shRNA directed against PC1/3 labeled with (A) early endosome marker EEA1 or RAB5. (D) baso-lateral membrane transport marker RAB8 and (F) late endosome/lysosome marker RAB7. In (A), arrows indicate punctate labeling near the plasma membrane. Insets represent 3× magnification. (B–C, G) EEA1 (B), RAB5 (C) and RAB7 (G) labelled vesicles area was estimated using ImageJ software. Vesicles where considered as ovals and area was calculated using the following formula: π×a×b where a and b are the two largest diameters. E. The ratio between peripheral and total integrated intensity of RAB8 labeling is represented. H. RAB7 peri nuclear integrated intensity is represented. * = 0.05 ** = 0.001 ***<0.0001, p-values, Student’s t-test. Quantification was performed on randomly selected field of view on two independent shRNA cell lines n = 4–6.

From our previous study, one of our most interesting observations using isolated macrophages derived from PC1/3 KO mice was the drastic change in the number, shape and density of vesicles [12]. This observation, made using transmission electron microscopy (TEM), is indicative of several events that affect vesicle trafficking. We therefore conducted a confocal microscopy study to determine whether the down-regulation of PC1/3 would affect vesicle trafficking markers in NR8383 cells. Little or no difference was observed in the recycling vesicle marker RAB11 [54], [55], the lysosome to TGN recycling marker RAB9 [56], [57] and the TGN marker TGN46 [45], [46] (**sup. data Figure S4**). However, we observed notable changes in the early endosome markers EEA1 [47], [48] and RAB5 [47], [58] (**Figure 7A–C**), the basolateral transport marker RAB8 [59]–[61] (**Figure 7D–E**) and the lysosomal marker RAB7 [62], [63] (**Figure 7F–H**).

**Figure 8:**
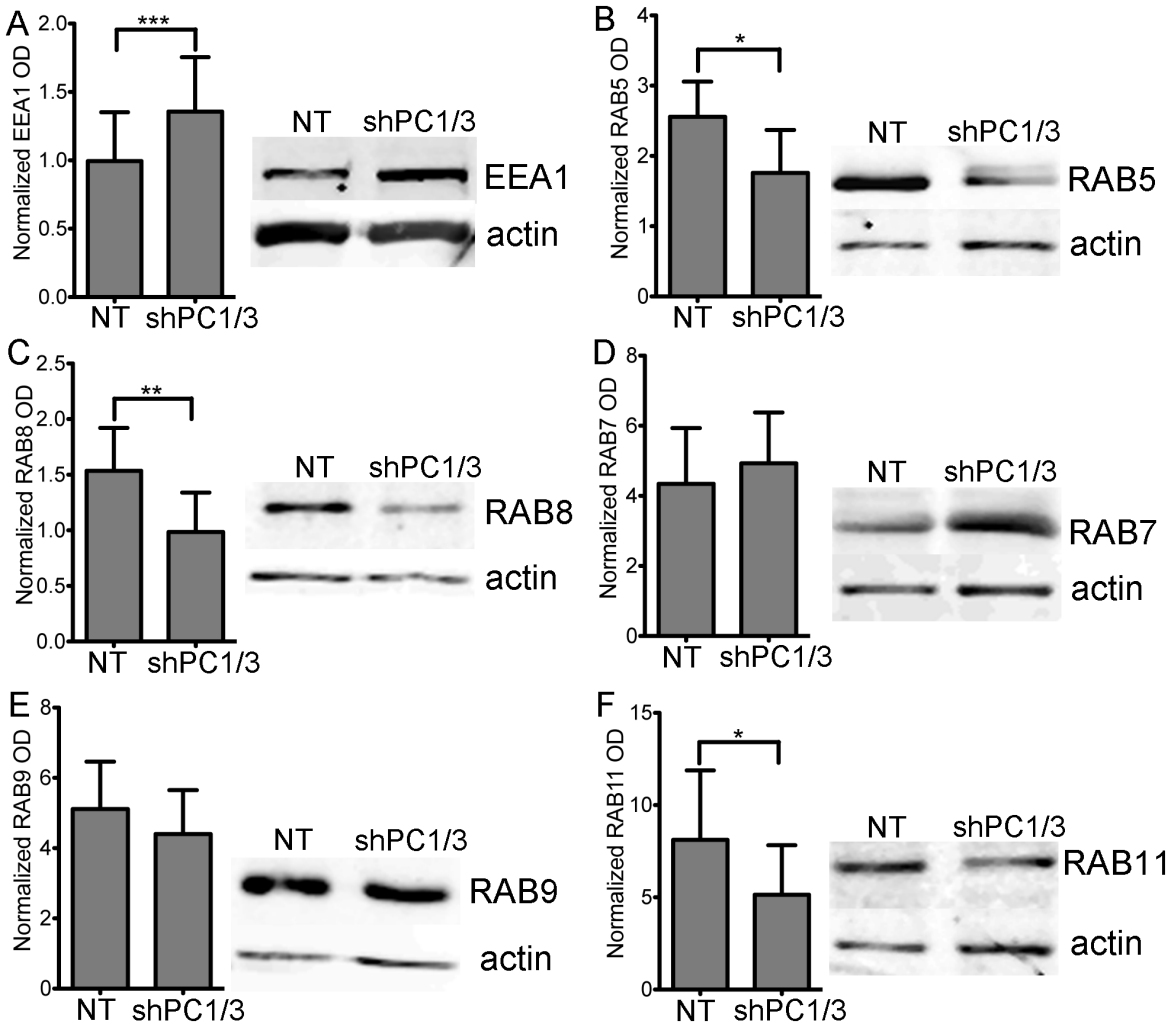
Effects of PC1/3 down-regulation on vesicle trafficking markers protein expression. Representative western blots (20 µg of protein) and gels optic density (OD) quantification showing the relative levels of vesicle trafficking markers EEA1 (A), RAB5 (B), RAB8 (C), RAB7 (D), RAB9 (E) and RAB11 (F) between NR8383 cells expressing control shRNA (NT) and those expressing shRNA directed against PC1/3 (SH). The actin loading control is included. * = 0.05 ** = 0.001 ***<0.0001 p-values, Student’s t-test n = 5–8.

In non-target (NT) shRNA treated NR8383 control cells, the early endosome markers EEA1 and RAB5 labeled the TGN region and areas near the PM (**Figure 7A** **).** RAB5 labeling was stronger near the PM, and EEA1 labeling was stronger near the TGN region. Both markers indicated the formation of medium-sized vesicles (**Figure 7A** **, inset**). In shPC1/3 cells, one of the most important observations was the increased number of EEA1 and RAB5 labeled medium-sized vesicles. We also observed an increase in vesicle size in shPC1/3 cells vs NT shRNA controls (0.68±0.08 vs 0.47±0.05 µm^2^ for EEA1 **Figures 7B** and 1.5±0.3 vs 0.28±0.05 µm^2^ for RAB5 **Figure 7C** **,** respectively). Although both markers presented a more diffuse distribution, a higher density of vesicles forming near the PM was observed (**Figure 7A**, **arrows**). When we quantified the integrated optical densities of EEA1 and RAB5 (pixel intensity x area), we observed no significant difference between NT shRNA and PC1/3 shRNA. This means that the overall cellular signals are comparable. However, mean pixel intensities for EEA1 were lower in PC1/3 shRNA than NT shRNA cells (42±3 vs, 66±8, respectively, p = 0.009 Student’s t-test). In addition, there was an increased peripheral labelling in PC1/3 shRNA compared to NT shRNA cells (33±6% vs 22±4%, respectively, p = 0.01 Student’s t-test). Together these data are indicative of a broader distribution of EEA1 labeled endosomes, but also of their closer proximity to the PM. We also verified tested the expression levels of EEA1 (**Figure 8A**) and RAB5 (**Figure 8B**) by western blotting and observed an increase in EEA1 expression but a decrease in RAB5 expression.

RAB8 labeling was mostly present at the basal PM of NT shRNA control cells, exhibiting tubular labeling and some vesicle-like structures (**Figure 7D**, **inset**). RAB8 also labeled the TGN/Golgi region. In shPC1/3 cells, the tubular labeling at the basal PM was more intense, accompanied with an increase in tubular structures/cells compared to NT shRNA cells (9±3 vs 2±1 tubules/cells, respectively, p = 0.008 Student’s t-test). Additionally, many cells showed a strong and dense labeling of small vesicle-like structures near the PM as well as decreased and diffuse labeling of the TGN/Golgi. The latter observation was confirmed by an increase in the peripheral labeling of RAB8 in shPC1/3 cells as compared to NT shRNA controls (34±1 vs 22±4%, respectively, **Figure 7E**). This observation can be explained by the decreased RAB8 expression in shPC1/3 cells demonstrated by western blotting (**Figure 8C**). Thus, we can conclude that PC1/3 down-regulation affects basolateral trafficking.

RAB7 exhibited the most intense labeling of all RAB proteins observed in NR8383 cells and was distributed throughout the cell with the strongest labeling near the TGN/Golgi region (**Figure 7F**). RAB7 also labeled large vesicle-like structures (**Figure 7F**, **inset**). In shPC1/3 cells, RAB7 labeling was even more intense in the perinuclear region (**Figure 7H**), where it appeared in multiple large and well-formed vesicles (**Figure 7F**, **inset**). These changes were quantified showing an increased vesicle size (**Figure 7G**). Some weakly labeled large vesicles were also observed near the PM. By western blotting, RAB7 expression was shown to be unchanged (**Figure 8D**). Thus PC1/3 down-regulation also has an impact on lysosome formation and trafficking.

Our analysis of RAB proteins and EEA1 leads us to the conclusion that PC1/3 down-regulation results in dramatic changes in cellular structures and trafficking in NR8383 cells. These results agree with our previous data obtained in isolated PC1/3 KO peritoneal macrophages [12].

### PC1/3 Down-regulation Modulates Cytokine Secretion in NR8383 Cells

**Figure 9:**
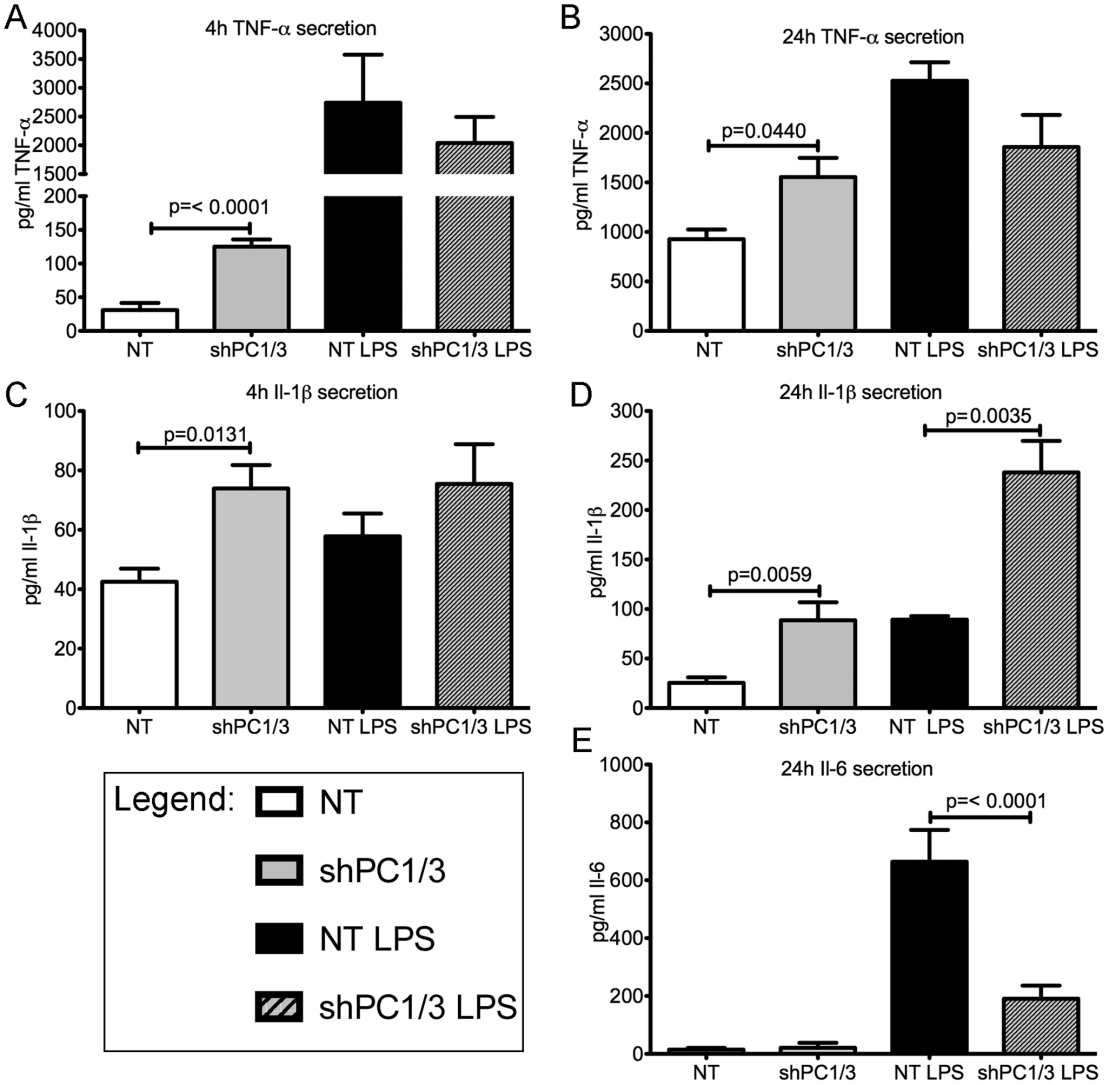
Effects of PC1/3 down-regulation on cytokine secretion in NR8383 cells. Cytokine secretion measured by ELISA in control NR8383 cells expressing control shRNA (NT) and shRNA directed against PC1/3 in culture media with or without LPS stimulation (100 ng/mL) at various time points. A. TNF-alpha 4 h, B. TNF-alpha 24 h, C. IL-1beta 4 h, D. IL-1beta 24 h and E. IL-6 24 h. p-values were obtained using Student’s t-test (n = 4–6).

In PC1/3 KO mice, an innate immune challenge results in the massive secretion of cytokines that is related to macrophage dysfunction [12]. Thus we aim to determine whether PC1/3 shRNA would induce a similar phenotype in the NR8383 cell model. We observed a 4-fold increase in basal TNF-α secretion after 4 h and a 1.6-fold increase after 24 h incubation in shPC1/3 NR8383 cells compared to NT shRNA cells (**Figure 9A–B**). LPS stimulation did not affect TNF-α secretion when compared with the control condition. However, the fold changes in TNF-α secretion induced by LPS stimulation (LPS/basal) was reduced in PC1/3 shRNA cells, most likely as a consequence of increased basal secretion levels. For the inflammasome-related cytokine IL-1β [64], a similar increase in basal secretion after 4 h (1.7 fold) and 24 h (3.5 fold) incubation was observed in PC1/3 shRNA down-regulated NR8383 cells (**Figure 9C–D**). However, a 2.7-fold increase in IL-1β secretion after 24 h of LPS stimulation was detected in PC1/3 shRNA down-regulated cells, which is equivalent to the 3-fold increased secretion compared to LPS induced control cells. For IL-6, basal secretion was very low, at the limits of detection after 4 h and 24 h incubation (**Figure 9E**), as well as secretion after 4 h of LPS stimulation (not shown). However, after 24 h of LPS stimulation, there was a 3.5-fold decrease in IL-6 secretion in PC1/3 down-regulated cells compared to control cells (**Figure 9E**). We also examined other inflammatory cytokines such as IL-12p70 [65] and regulatory cytokine IL-10 [66]; however, we failed to observe reproducible and detectable levels of either cytokine (data not shown). Taken together, these results demonstrate important changes in basal cytokine secretions in PC1/3 shRNA down-regulated NR8383 cells, indicative that PC1/3 strongly affect cellular trafficking events.

## Discussion

PC1/3 plays a critical role in the neuroendocrine system, and our knowledge-base regarding PC1/3 has benefited from the study of several model cell lines, notably AtT-20 [67] and β-TC3 cells [42]. The discovery of a human PC1/3 deficiency [68] and the generation of the PC1/3 KO mouse model [69] have further defined the PC1/3 phenotype. In a recent study using the same PC1/3 KO mouse model [12], we established a novel role for PC1/3 in the maintenance of immune homeostasis, which adds to the growing body of evidence indicating a role for PC1/3 in macrophages [9], [11], [70]. Thus, elucidation of the cellular biology and biochemistry of PC1/3 in macrophages is relevant and we report here that NR8383 cells are a suitable cellular model. NR8383 cells express TLRs and were previously shown to be LPS- [24] and Poly:IC-responsive [41]. NR8383 cells express PC1/3 (mRNA and protein), the known PC1/3 regulator proSAAS [71], [72], but not PC2. These finding correlate well with the profile previously reported in rat-isolated macrophages [11]. Interestingly, the widely used RAW 264.7 cells did not express PC1/3 (data not shown). RAW264.7 cells are derived from ascites [73] and NR8383 cells are more similar to resident alveolar macrophages [33]. Another human monocyte/macrophage cell line, THP-1, was previously shown to express PC1/3 [10], but THP-1 cells need to be induced with PMA to differentiate into macrophages. We believe that PMA treatment may introduce variability to our studies; thus, it would be difficult to predict the stability and efficiency of knockdown strategies such as shRNA.

PC1/3 traffics in neuroendocrine cells [74]–[77] within the regulated secretory pathway, but in macrophages this pathway is not conventional [51], [52], [78]–[80]. Thereby, the present report highlights marked differences in PC1/3 cellular distribution when compared with neuroendocrine cells. In NR8383 cells, PC1/3 was mostly retained at the TGN in a pool that translocates to LAMP1-related vesicles. This pool was especially visible when macrophages exhibited phagocytic-like activities or when the cells where LPS stimulated. PC1/3 did not appear to accumulate in specialized secretory vesicles. We thus conclude that the TGN is where PC1/3 is primarily retained in NR8383 cells. EM confirmed these data and showed that PC1/3 was widely distributed in NR8383 cells with a preference for ER, TGN, phagolysosomes and late endosome vesicles. It is noteworthy that LPS triggers PC1/3 exit from the TGN toward LAMP1-labeled vesicles, as well as a co-localization with the LPS receptor TLR4. The expression of PC1/3 in macrophages [11] and in immune-associated tissues [9] was already shown to be LPS-sensitive. The novel LPS-triggered trafficking of PC1/3 is what most closely resembles the well-known stimulus-dependent PC1/3 secretion and could be regulated by the phospho-mannose receptor pathway directed by the phosphorylation of mannose moieties in the N-glycosylation sites of PC1/3 [81]. LPS stimulation is known to induce dramatic changes in the organization of organelles within macrophages [53], [82], [83]. The observed intracellular reorganization of the TGN46 was confirmed in our study, but it remains unclear whether LPS-induced PC1/3 trafficking is tightly regulated or is simply part of a general cell reorganization mechanism.

The stimulus-dependant trafficking of PC1/3 suggests a functional role in the cellular compartments to which it is localized and suggests other questions as to whether it is LPS-specific or whether other TLR ligands could induce similar changes in its localization. The change in localization of PC1/3 from the TGN toward LAMP1-labeled compartments provides an important hint. For example when PC1/3 reaches acidic vesicles, it would be converted to shorter and more active forms [84]. Moreover, lysosomes and phagolysosomes are well known to be a part of the secretory pathway in macrophages [51], [52], [78]. Thus, this trafficking event may imply changes in PC1/3 activity that could affect paracrine roles involved in an immune response. So far, we have only detected full-length PC1/3 in our experiments. In “granule rich” like fractions from THP-1 stimulated cells, the shorter forms of PC1/3 (ΔCT PC1/3) were observed [10], as well as in isolated rat alveolar macrophage and spleen monocytes [11]. Unfortunately, PC1/3 does not heavily concentrate in storage compartments in macrophages, as it does in the secretory granules of neuroendocrine cells. This combined with the lack of sensitivity of our western blots prevented us from obtaining more detailed data for the characterization of PC1/3 processing in NR8383 cells. Further studies, in the context of our present trafficking results will be required to elucidate this question.

Using shRNAs we were able to validate our previous observation obtained in macrophages isolated from PC1/3 KO mice [12]. An unexpected phenotype was observed in PC1/3 KO mice revealing a deregulation of the LPS-induced secretion of pro-inflammatory cytokines. This deregulated cytokine secretion was also observed in isolated PC1/3 KO macrophages. Here, we report somewhat similar findings in NR8383 cells following the down-regulation of PC1/3, where the basal secretion of TNF-α and IL-1β was greatly increased, while 24 h of LPS stimulation drastically increased IL-1β secretion. It was also observed that 24 h of LPS stimulation had no further effect on TNF-α secretion but reduced IL-6 secretion. These differences can be explained by the time course used to observe differences in cytokine secretion and the specific cytokines that exerted these effects in NR8383 shPC1/3 cells differ from what was reported for macrophages isolated from PC1/3 KO mice. Additionally, the purity of the peritoneal extract, the long-term potentiation of peritoneal macrophages by other immune cells and the origin of the macrophage itself may explain these disparities. Still, when regarding the global effects on cytokine secretion, it is clear that PC1/3 plays an important role in the control of cytokine secretion in macrophages.

Our previous EM studies on isolated peritoneal macrophages from KO mice explain in part why cytokine secretion was deregulated. These studies uncovered drastic changes in the cellular organization of PC1/3 KO macrophages [12]. In macrophages, constant transformation occurs between tubuloreticular and vesicular formations [85]. This process involves ligand-receptor binding, intricate signal transduction networks, focal cytoskeleton rearrangement and a dynamic series of membrane fusion/fission and remodelling events [86]. We investigated the effect of PC1/3 down-regulation on these processes. NR8383 shPC1/3 cells showed expression changes and intracellular re-organization of RAB7-, RAB8- and EEA1/RAB5-related markers. Interestingly, the effects seemed to be constrained to specific markers and vesicles because no effects were observed for RAB9, RAB11 or TGN46. These results are highly similar to previous findings in isolated KO macrophages and are indicative of the regulatory mechanisms of cytokine secretion. Thus, we propose two hypotheses to explain these changes. First, PC1/3 may affect the activity of a substrate through its processing and trigger signalling events that could regulate the expression and activation of RAB proteins. It was previously reported that substrates of PC1/3 are secreted by macrophages, notably proopiomelanocortin and somatostatin [10], [11], [70], [87], [88]. Given the cellular localization of PC1/3, it may even play a role within the phagocytic synapse, where several signalling events are initiated [89], [90]. A second hypothesis would imply that PC1/3 could act as a binding partner or a possible chaperone within the secretory pathway. It has been proposed that PC1/3 participates in protein sorting to DCSGs by binding to the recognition cleavage site composed of paired basic amino acids [43], [91]–[93]. Knockout models have revealed that in absence of PC1/3 some neuropeptide substrates tend to be overrepresented [94]. Thus, without the need for processing a specific substrate, PC1/3 could use paired basic amino acids as a sorting motif and could regulate several trafficking events.

The characterization of the rat alveolar NR8383 cell line shows great promise as a model cell line to study the role of PC1/3 in innate immunity and is a step toward achieving a better understanding of this non-conventional role of PC1/3. Future studies on the expression of PC1/3 substrates as well as activation and trafficking regulation in NR8383 cells will provide clues regarding how the knockout of PC1/3 causes a global deregulation of the innate immune response.

## Supporting Information

Figure S1 **Alignment between PC1/3 RT-PCR product from NR8383 and sequence of rat NR8383 NM_017091.**
(EPS)

Figure S2 **TLR4 and PC1/3 (using N-terminal antibody) co-localize during LPS stimulation.**
(TIF)

Figure S3 **PC1/3 and EEA1 do not co-localize during LPS stimulation.**
(TIF)

Figure S4 **PC1/3 shRNA in NR8383 do not affect RAB9, RAB11 and TGN46 distribution.**
(TIF)
