# Expression of Concern: Proprotein Convertase 1/3 (PC1/3) in the Rat Alveolar Macrophage Cell Line NR8383: Localization, Trafficking and Effects on Cytokine Secretion

**DOI:** 10.1371/journal.pone.0311702

**Published:** 2024-10-02

**Authors:** The PLOS ONE Editors

After this article [1] was published, concerns were raised about Figs 1D and 2.

Specifically:

- In Fig 1D, similarities were noted between the bands in lane 1 of the PC1/3 panel and lane 2 in the 18S panel, and between lanes 1 and 3 in the 18S panel. There are vertical discontinuities between lanes 1 and 2 in both PC1/3 and 18S panels. There also appears to be a horizontal discontinuity below the band in lane 1 of the PC1/3 panel, and the regions below the bands in lane 1 and lane 2 of the PC1/3 panel appear similar.
- In Fig 2B, there appear to be horizontal discontinuities below the upper bands in the NT and shPC1/3 lanes.
- In Figs 2B and 2C, there appear to be horizontal discontinuities between the 50 and 75 kDa markers, and vertical discontinuities between the AtT-20 and NT lanes.

The first author (HG) stated that the original image data underlying this article’s results are no longer available.

In response to the concerns, the first author stated that the Fig 1D blot images were spliced between lanes 1 and 2 to remove data that were not relevant to this publication, and that there are differences between the lanes for which similarities were noted. He also said, “*the published figure is the raw data*. *It was produced on film and numerized with a camera 20 years ago*. *You can see the numerization pixels that are different from one figure to the other (camera glitch)*. *The figure as it’s presented is the actual numerized raw data since it was numerized this way (cropped)*. *The camera glitch you can see*, *when you play with hue/saturation and contrast*, *actually proves that each one of the bands is coming from a different numerization*.” The *PLOS ONE* Editors remain concerned that the indicated regions of Fig 1D appear more similar than would be expected for independent results.

For Fig 2B, the last author (RD) stated that a specific region of the blot was selectively enhanced following a reviewer’s request to better show the PC1/3 bands, resulting in the horizontal discontinuity below the upper bands. The first author noted that the vertical splice lines in Figs 2B and 2C were introduced during figure assembly due to the high difference of expression between AtT-20 and NR8383 cells, which suggests that lanes 1 and lanes 2/3 in each of these panels may not be taken from the same image or exposure.

The first author also noted that the lower sections of the images in Fig 2B and 2C (below the splice line between 50 and 75 kDa) show actin blots, they should have been separated from the upper blot images and labelled accordingly.

The above concerns cannot be fully clarified without the original image data, and the author’s comments about selective enhancement of a blot area in Fig 2B raise further concerns about image data reporting. The *PLOS ONE* Editors issue this Expression of Concern to inform readers of these unresolved issues, although data reported in other panels appear to uphold the claims about PC1/3 mRNA expression in NR8383 cells (Fig 1) and reduced PC1/3 protein expression in shRNA-treated NR8383 cells (Fig 2).
