# Supplementary figures and images for: Proprotein Convertase 1/3 (PC1/3) in the Rat Alveolar Macrophage Cell Line NR8383: Localization, Trafficking and Effects on Cytokine Secretion
Source: PLoS One. 2013 Apr 24;8(4):e61557. doi: 10.1371/journal.pone.0061557 (PMC3634814; doi:10.1371/journal.pone.0061557)

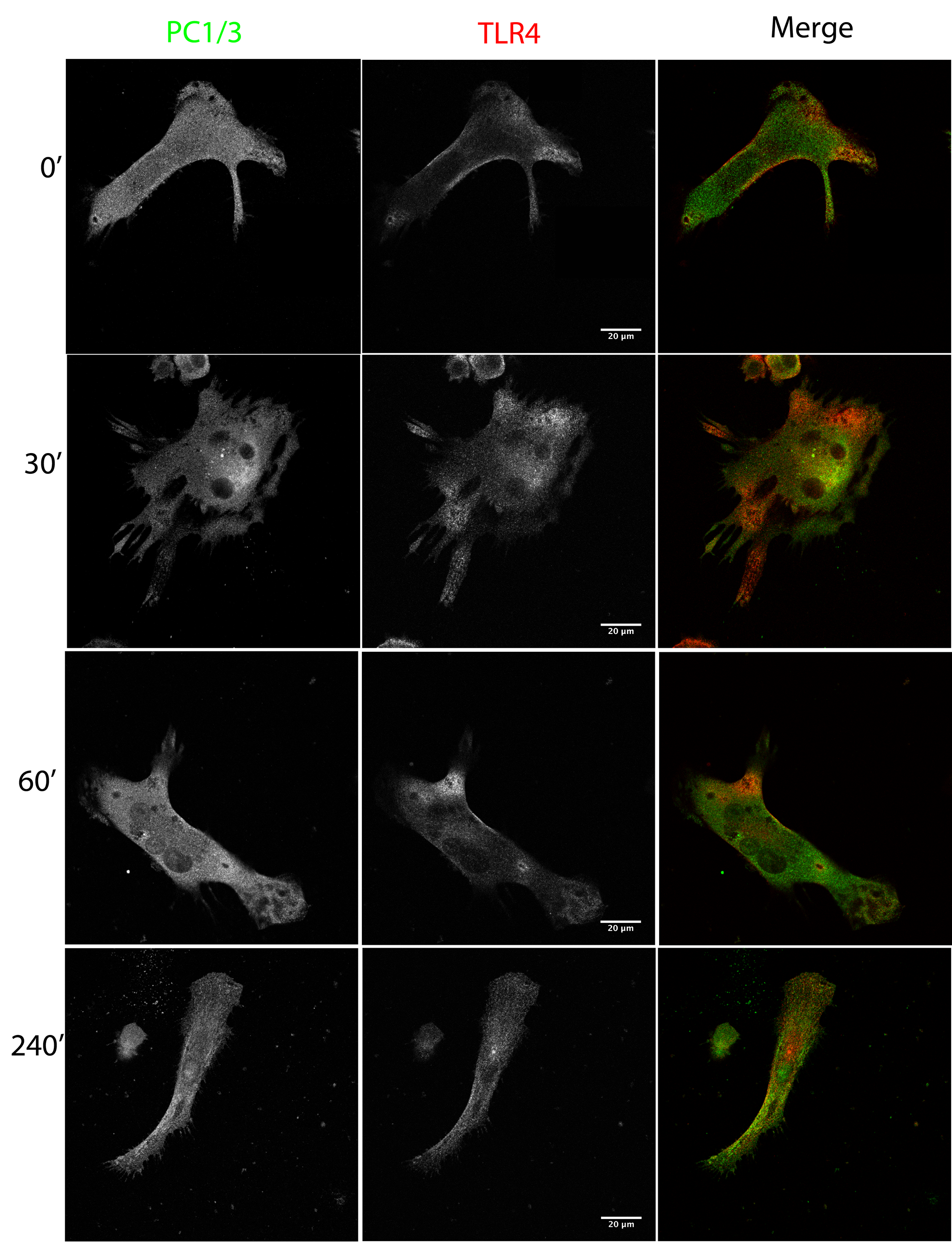

Supplement: Figure S2 — TLR4 and PC1/3 (using N-terminal antibody) co-localize during LPS stimulation. (TIF) [file pone.0061557.s002.tif]

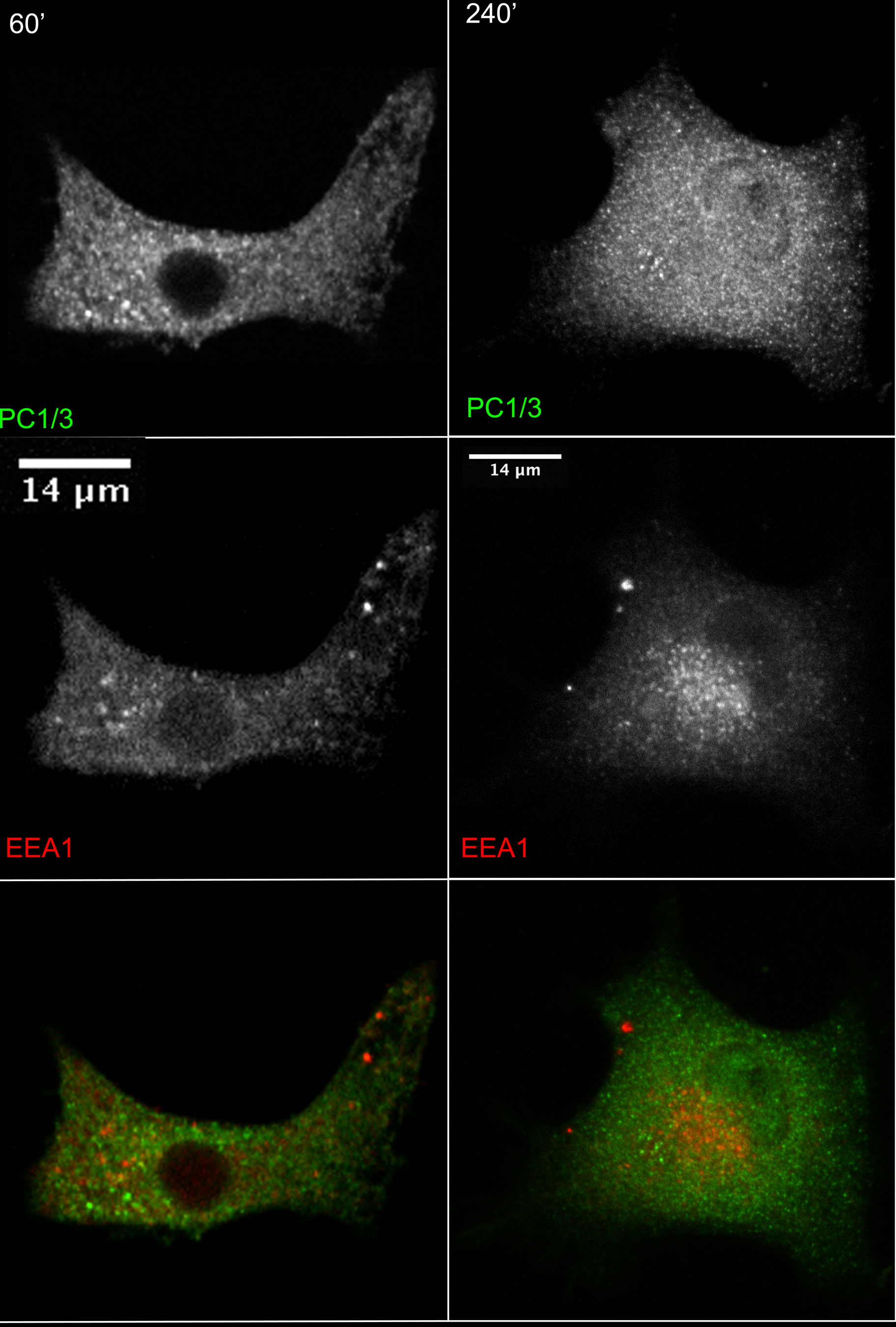

Supplement: Figure S3 — PC1/3 and EEA1 do not co-localize during LPS stimulation. (TIF) [file pone.0061557.s003.tif]

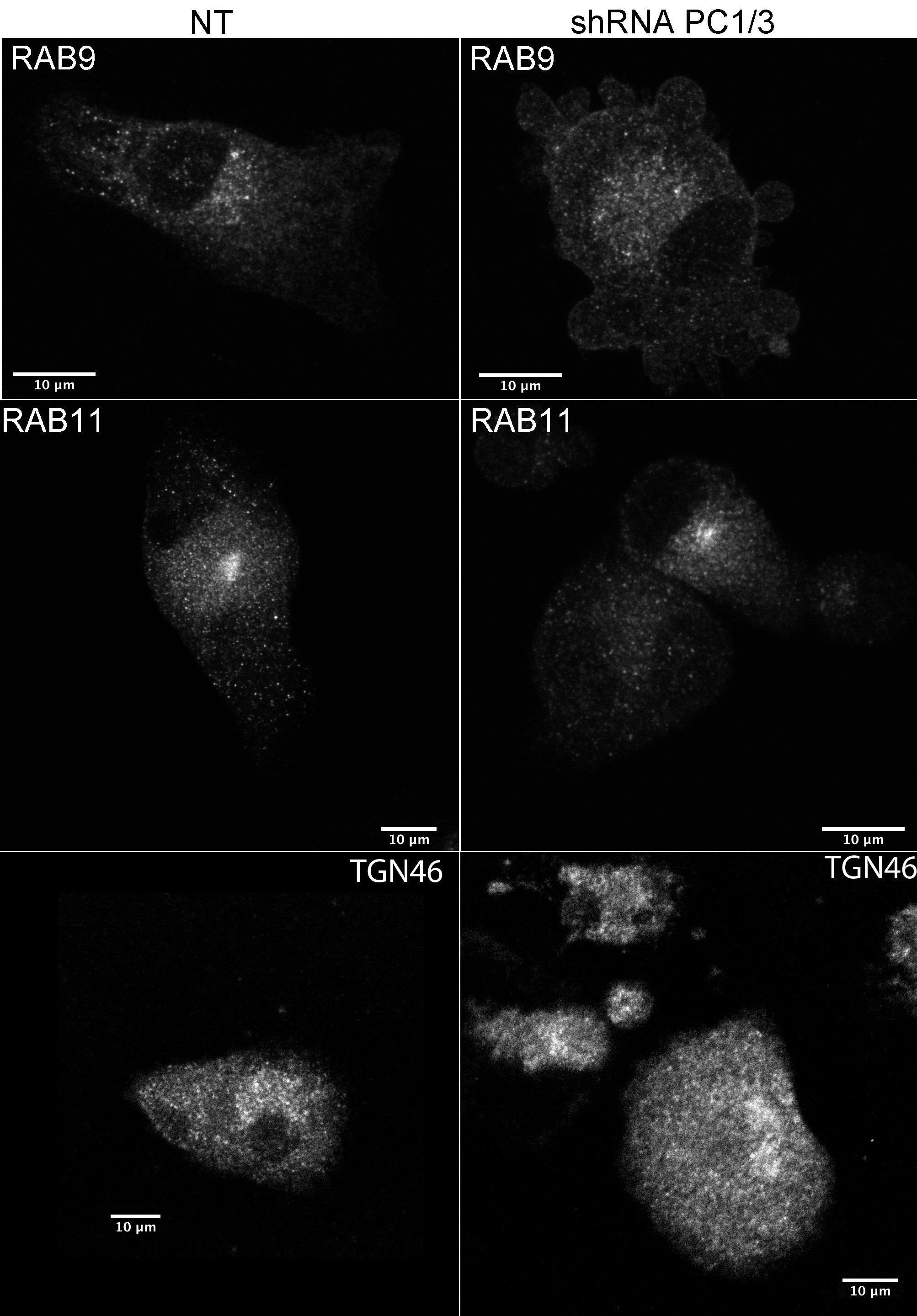

Supplement: Figure S4 — PC1/3 shRNA in NR8383 do not affect RAB9, RAB11 and TGN46 distribution. (TIF) [file pone.0061557.s004.tif]
